# Structural and intermediary determinants of social inequalities in the mental well-being of European workers: a relational approach

**DOI:** 10.1186/1471-2458-14-938

**Published:** 2014-09-09

**Authors:** Deborah De Moortel, Hadewijch Vandenheede, Carles Muntaner, Christophe Vanroelen

**Affiliations:** Department of Sociology, Vrije Universiteit Brussel, Pleinlaan 2, 1050 Brussel, Belgium; Health Inequalities Research Group, Employment Conditions Knowledge Network (GREDS\Emconet), Universitat Pompeu Fabra, Plaça de la Mercè 10-12, 08002 Barcelona, Spain; Bloomberg Faculty of Nursing, University of Toronto, Toronto, ON M5T 1P8 Canada; College of Health Sciences, Korea University, Seoul, 136-703 Korea

## Abstract

**Background:**

The objective of this study is to examine social inequalities in employee mental well-being, using relational social class indicators. Relational social class indicators are based on theoretical insights about the mechanisms generating social (health) inequalities. Additionally, it is examined whether the psychosocial work environment and employment quality act as intermediary determinants of social class inequalities in mental well-being, simultaneously testing the mediation (differential exposure) and moderation (differential vulnerability) hypotheses.

**Methods:**

Data from the European Social Survey Round 2 (2004/5) and Round 5 (2010) were analysed. Mental well-being was assessed by the WHO Well-being Index. The measure for social class was inspired by E.O. Wright’s class scheme. Three-level linear multilevel modelling was used to account for clustering of employees within research years and countries.

**Results:**

We found social class inequalities in mental well-being in the European working population for both men and women. Compared to unskilled workers, managers reported the best mental well-being, while supervisors held an intermediary position. As regards the mediation hypothesis, an unfavourable psychosocial work environment and low-quality employment conditions mediated the relation between social class and poor mental well-being in both men and women. However, low quality of employment relations only mediated the “social class-mental well-being” association in the male sample. As regards the moderation hypothesis, modification effects were seen for the psychosocial work environment and employment conditions in both men and women.

**Conclusion:**

Relational indicators of social class are related to mental well-being in European employees. Relational accounts of social class are complementary to stratification indicators in social epidemiology. From a policy perspective, better employee mental well-being and less social class inequality could be achieved through initiatives addressing the unequal social relations generated by structural positions in the labour process.

## Background

Many studies have shown social gradients in physical ill health and mortality among employees across Europe: the lower workers’ socioeconomic position, the higher their risk of poor health or mortality [1–3]. Social gradients among workers are also seen for serious mental illnesses and major depression [2, 4]. However, the socioeconomic patterning of less severe mental health problems is less clear [5].

Nevertheless, workers who are free from mental illness are not necessarily in good mental health [6]. Mental health is more than the absence of mental illness, but includes also a reflection of the presence of positive feelings and positive functioning in life [6, 7]. The absence of positive feelings about life, such as feelings of stress or anxiety, is debilitating for the individual in various ways [8]. Research shows that these minor mental health problems among employees are an important cause of sickness absence and work disability [9]. Hence, their burden on society is quite heavy [8].

Studies investigating social inequalities in less severe mental health problems among employees show inconsistent results: some report a better mental health or well-being for employees in lower socioeconomic positions, while other studies find the associations to be statistically insignificant [10–13].

One of the reasons for contradictory findings might lie with the measures for socioeconomic position most researchers are using. Often they have no explicit underlying theoretical framework [14, 15]. In empirical social epidemiological research, socioeconomic position is commonly measured as “social stratification” - that is, as a gradient based on for instance income or years/grades of education [16]. These measures typically do not reveal the social mechanisms that explain how individuals accumulate different levels of material and psychosocial resources [17]. Unlike measures of social stratification, relational social class indicators are based on theoretical insights about the mechanisms generating social (health) inequalities. Among workers, unequal social relations are generated by structural positions of dominance and subordination in the labour process. It can be assumed that these unequal relations are the core processes generating mental health inequalities. The social class approach represents thus a framework complementary to social stratification, tapping into parts of social health variations not captured by conventional measures of social stratification [18].

Another reason for contradictory findings might be the limited adequacy of a gradient approach for the identification of minor mental health problems related to specific “roles” in the production process. Previous research found better mental health in workers without supervisory authority than in low-level supervisors due to the special structural position of supervisors [16]. Supervisors are subjected to the pressure of upper management’s control, while having responsibilities over subordinate workers. At the same time they exert little influence over company policy. In other words, because of their social role in the labour process, supervisors may have particular difficulties to deal with high demands and low control at work [16]. This finding gives support to Wright’s “contradictory class location” hypothesis [19]. It can be assumed that the “contradictory class location” of supervisors is likely to result in poorer mental health, whereas lower classified workers are likely to have better mental health. Descriptive, gradient-wise indicators may “hide” less favourable mental health outcomes of higher classified groups [20].

First, more research is needed on social inequalities in minor mental health problems in European employees, using a relational social class approach and taking workers’ specific roles into the production process into account. In this study mental well-being (as measured by the WHO Well-being Index) will be used as an indicator of minor mental health problems. High mental well-being is increasingly recognised by policy makers and health advocates as an indicator of good mental health [21]. Second, the role of contemporary work and employment arrangements in the relationship between social class and employees’ well-being should be explored. Previous research showed that exposure to adverse work and employment characteristics is mediating (explaining) the association between social class and self-reported health: workers in less empowered, more exploited classes are more frequently exposed to adverse work and employment characteristics, than workers in more empowered, less exploited classes [18]. However, adverse work and employment exposures could also moderate the relation between mental well-being and social class, in the same way as these exposures have shown to moderate the relation between measures of social stratification and health outcomes [22]. In other words, workers in less empowered, more exploited classes could be more vulnerable to adverse work and employment characteristics, resulting in worse mental well-being, compared to more empowered, less exploited classes. Both differential vulnerability and differential exposure of employees to adverse work and employment characteristics should be examined when studying social inequalities in mental well-being.

### Structural and intermediary determinants of mental health inequalities

Mental health inequity has structural and intermediary determinants in which structural determinants come first in the causal process [23, 24]. Structural determinants of health inequalities are social, economic and political mechanisms which generate social class inequalities in society [24]. Social class can be considered a structural determinant of mental health, while situational characteristics (such as job quality) can be considered as a mechanism interfering with the relation between social class and mental health.

According to Wright [25], employees sell their labour power to the owners of productive assets (employers) who extract labour effort from them. Some employees, such as managers and supervisors, receive delegated authority from employers: they have the power to supervise subordinates and give them positive and negative sanctions. Managers and supervisors dominate (hire, fire, promote, demote) subordinates, but are controlled by employers themselves. This is why Wright [25] argues that supervisors and managers occupy a contradictory location within class relations. Managers and supervisors also differ from each other: managers are able to influence organisational decision-making, while supervisors are not [25]. Furthermore, employees who possess high levels of skills (so-called “experts”), are potentially in a privileged appropriation location within the class structure, because their specific skills or expertise are highly valued on the labour market [25]. Emphasising individuals’ location within these structural sets of relations calls attention to the on-going tensions between managers, supervisors and workers with different levels of skills and expertise, generating social inequalities in mental health [19]. A Spanish study found that the repeated experience of organizational control at work protected most upper-level managers against mood and anxiety disorders. This study also showed that low-level supervisors reported higher rates of depression and anxiety than both high-level managers and non-supervisory workers. Moreover, experts were found to enjoy better health than non-experts [16]. We hypothesize that in the European employee population, compared to workers, managers report better mental well-being, while supervisors present worse mental well-being. We furthermore expect experts to report better mental well-being than non-expert employees.

The position in the class structure also shapes specific determinants of health status [24]. As mentioned earlier, based on their class position, individuals experience differences in exposure and vulnerability to health-compromising conditions [26, 27]. These conditions, such as unfavourable working conditions, operate as intermediary determinants of health inequalities. In empirical studies, intermediary determinants of health inequalities among workers are usually assessed through indicators of the psychosocial work environment [2, 28]. For instance, using the Demand-Control model: high psychological demands have a detrimental effect on mental health, while high job control is related to better mental health [29]. However, compromised employment quality is also found to be negatively associated with mental health [30]. Employment quality encompasses two conceptual dimensions: employment conditions (contract security, working times, income and rights, and employability) and employment relations (empowerment and representation) [31]. Both studies on employment conditions and studies on employment relations found negative associations with workers’ well-being [32, 33]. We hypothesize that the association between social class and mental well-being can partially be explained by differential exposure (the mediation hypothesis) and differential vulnerability (the moderation hypothesis) to the psychosocial work environment, employment conditions and employment relations.

## Methods

### Data

This study is based on secondary data, namely on a pooled dataset of Round 2 (2004/5) and 5 (2010) of the European Social Survey (ESS). In these rounds, the questionnaire contained a supplementary module on family, work and well-being. Due to the exclusive use of secondary data (ESS data), which is available to the public, no ethical approval is required for this study. The ESS includes representative samples of persons aged 15 and over, who are resident in European countries. This study focuses on European wage earners. Respondents not in waged employment or older than 65 were excluded from the analyses. In addition, only data from countries that participated in both ESS rounds were retained. This left us with data from 19 European countries (Belgium, Czech Republic, Denmark, Estonia, Finland, France, Germany, Greece, Hungary, Ireland, Netherlands, Norway, Poland, Slovakia, Slovenia, Spain, Sweden, Switzerland, and United Kingdom) to analyse, and a total sample of 15,030 male and 14,683 female employees. For the multivariate analyses, we applied complete case analysis reducing the number of respondents to 14,583 male and 14,164 female employees.

### Measures

For all “scale” variables goes that they were normalised to range from 0 to 10, with 10 being the least-favourable situation [34]. Whenever an item was missing on the scale or the combined variables, this item was attributed a value using expectation-maximisation as imputation method [35].

### Social class

The indicators of social class were inspired by Wright’s social class scheme and were obtained through the combination of the International Standard Classification of Occupations (ISCO), the International Standard Classification of Education (ISCED), and a question on whether the employee is responsible for supervising other employees. First, three categories were created: managers, supervisors and workers. Employees in occupational group ISCO 1 were considered managers as these occupations are characterised by a high level of organisational control. Non-managers who reported to be responsible for supervising other employees were considered supervisors. All other employees were considered workers. Second, within these three categories, another subdivision was made using ISCED: “unskilled” (up to lower secondary); “semi-skilled” (up to post-secondary non-tertiary); and “experts” (completed tertiary education). Because of sample size limitations, in the multivariate analyses, unskilled and semi-skilled supervisors on one hand, and unskilled and semi-skilled managers on the other were pooled together (resulting in non-expert supervisors and non-expert managers respectively).

### Poor mental well-being

Poor mental well-being was measured by three items from the WHO-5 Well-being Index [36]. The WHO-5 Well-being Index is a measure of positive affect [37]. The ESS 2010 only contained three of the original five items of the WHO-5 Well-being Index [36]. However, the internal consistency of the Well-being Index has proven to be excellent. The three items have a Cronbach’s alpha of 0.81 across the whole ESS 2010 sample and a Cronbach’s alpha of 0.78 across the study sample, which is only marginally lower than the Cronbach’s alpha of 0.82 found across the whole ESS 2004 sample which contained all five items from the WHO-5 Well-being index [36]. Consequently, we can be confident that the use of the three-item scale does not lead to different results. The questions included were: (1) over the last two weeks I have felt cheerful and in good spirits, (2) over the last two weeks I have felt calm and relaxed, and (3) over the last two weeks I have felt active and vigorous [37]. Answers range from (1) “All of the time” to (6) “At no time”. The item scores were summed and then normalised to a 0 to 10 range.

### Psychosocial work characteristics

The psychosocial work characteristics were measured by the Demand-Control model [29]. Sum scales for low skill discretion and low autonomy were created. *Low skill discretion* is measured by three items: (1) variety in work; (2) job requires learning new things; and (3) how long for somebody with the right qualifications to learn to do your job well. The *low autonomy* scale also consists of three items: (1) allowed to decide how daily work is organised; (2) can decide time start/finish work; and (3) allowed to choose/change pace of work. *High psychological demands* are based on a five-point Likert item: “I have never enough time to get everything done in my job”.

### Employment quality

A multidimensional construct was created to assess employment quality (encompassing employment conditions and relations) [31].

Five indicators were selected to reflect the four dimensions of the quality of employment conditions: contract type, income, irregular and/or unsocial working hours, employment status, and lack of training. *Contract type* is a categorical variable distinguishing between “permanent contract”, “non-permanent contract” and “no contract”. *Income* is measured by combining two questions: a question on the perception of the current household income being sufficient (or not) and a question on the proportion of household income the respondent provides for (main or contributory earner). Measuring income sufficiency at the household is appropriate, since income - although related to individual employment situations - is a concern mostly situated at the household level. The income indicator consists of three categories: “sufficient household income”; “contributory earner with insufficient household income”; and “main earner with insufficient household income”. *Employment status* is a categorical variable distinguishing between “full-time”, “part-time” and “involuntary part-time” employment. The involuntary nature of part-time employment is included to control for personal preferences of the employees. Involuntary part-time employees are the respondents who work part-time (≤35 hours), but wish to work more hours. *Irregular and/or unsocial working hours* is measured by 4 items: (1) working weekends; (2) working evenings/nights; (3) working overtime at short notice and (4) intensive working hours. As a first step, a scale for unsocial hours was created, combining “working weekends” with “working evenings and nights” (Pearson correlation = 0.472 and Cronbach’s α = 0.640). Subsequently, the scale for unsocial working hours was added to the indicators for “working overtime at short notice” and “intensive working hours”, resulting in an overall scale for irregular and/or unsocial working hours (Cronbach’s α = 0.604). *Lack of training* is measured by a yes-no question on having been on a course for work during the last 12 months.

The quality of employment relations was measured by lack of co-worker support and lack of representation, reflecting the two dimensions of the quality of employment relations. *Lack of co-worker support* was assessed using the following question: “In current job: I can get support/help from my co-workers when needed”, and response categories were “not at all true”, “a little true”, “quite true” and “very true”. This indicator was dichotomised, with the category “not at all true” being considered as the low-support group. *Lack of representation* is measured through a yes-no question on the membership of a trade union or similar organisation.

In order to control for background characteristics, age was included in our statistical models. Age was recoded into three age groups: 15–29, 30–49 and 50–65. The age categories respond to the three main periods in a working career: lift-off (15–29 years), mid-career period (30–49 years) and end-of career period (50–65 years) [38].

### Statistical analyses

All analyses were sex specific. First, descriptive analyses were conducted. For all categories of the categorical variables, we determined the mean on the poor mental well-being scale. Differences in mean mental well-being between the categories were assessed with a series of one-way analysis of variance tests (ANOVA). For continuous independent variables, Pearson’s correlations were calculated between each variable and poor mental well-being. Throughout the descriptive analyses, data have been weighted by population weights correcting for population size and by design weights correcting for unequal selection chances. The descriptive analyses were performed using SPSS version 22.

In the multivariate analyses, three-level linear multilevel models were applied to statistically account for the clustering of the sampled employees within research years and countries. Individual employees at level-1 are nested within 38 country-year-cohorts at level-2, and country-year-cohorts are nested within 19 European countries at level-3. First, a three-level random intercepts model was estimated as a reference model, only including the individual background variable age. Second, indicators of social class were included in the reference model in order to estimate the distribution of poor mental well-being across social classes (Model 1). As this model only provides the differences between social classes as compared to the reference category (unskilled workers), we also fitted Model 1 extended by the interactions between skill level (unskilled, semi-skilled and expert) and social class (worker, supervisor and manager), obtaining similar results (not shown). Third, to test the mediation hypothesis (differential exposure), Model 1 was extended by indicators for the psychosocial work environment, the employment conditions and employment relations in Model 2, 3, and 4 respectively. Finally, to test the moderation hypothesis (differential vulnerability), Model 2, 3 and 4 were extended by the interactions between the indicators of social class and each set of intermediary determinants. All continous scales, except the poor mental well-being scale, were grand mean centered. At all steps, parameter effects of the covariates in relation with poor mental well-being are presented as coefficient estimates, with their related standard errors (SE). Multivariate analyses were carried out using Stata version 12.

## Results

### Description of the population

Table 1 provides the description of the study population, the mean poor mental well-being scores and the statistical differences between groups. The largest part of the population belonged to the class of semi-skilled workers (35.3% for women and 31.3% for men). We found marked gender differences in social classes (10.6% of men were managers compared to only 5.6% of women). The mean poor mental well-being score was highest among unskilled workers for women and among expert supervisors for men. Having no contract, an unfavourable income situation, holding an involuntary part-time job (for women), having a lack of training (for men) and a lack of co-worker support implied higher mean poor mental well-being scores. The mean poor mental well-being score was not statistically different for members and non-members of a trade union; therefore being a member of a trade union was omitted from the multivariate analyses. The psychosocial work environment was related to poor mental well-being. Low skill discretion (correlation is 0.07 for women and 0.05 for men), low autonomy (correlation is 0.05 for women and 0.03 for men) and high psychological demands (correlation is 0.07 for women and 0.11 for men) were positively, but weakly correlated to poor mental well-being. The correlation between irregular and/or unsocial working hours and poor mental well-being was also positive and weak (correlation is 0.03 for women and 0.05 for men).

**Table 1:** **Description of the population studied (number and percentages) and their average score on poor mental well-being (Population in salaried employment, 15–64 years old, ESS Round 2 and 5 (weighted))**

Variables	Women (n = 13,092)				Men (n = 13,928)			
	n	%	Mean poor mental well-being score	Sig.	n	%	Mean poor mental well-being score	Sig.
**Social class**				0.000				0.048
Workers unskilled	2067	15.8	3.9		1958	14.1	3.3	
Workers semi-skilled	4626	35.3	3.6		4363	31.3	3.4	
Workers experts	2875	22.0	3.5		1892	13.6	3.4	
Supervisors unskilled	296	2.3	3.4		584	4.2	3.2	
Supervisors semi-skilled	1105	8.4	3.4		2000	14.4	3.2	
Supervisors experts	1387	10.6	3.6		1652	11.9	3.4	
Managers unskilled	92	0.7	3.5		131	0.9	3.2	
Managers semi-skilled	247	1.9	3.8		458	3.3	3.4	
Managers experts	396	3.0	3.6		890	6.4	3.3	
**Age groups**				0.000				0.000
15-29 years	2494	19.1	3.4		2726	19.6	3.1	
30-49 years	7200	55.0	3.7		7527	54.0	3.4	
50 or more years	3398	26.0	3.7		3674	26.4	3.3	
**Psychosocial work characteristics**								
Low skill discretion^a^			4.5 (2.4)				4.1 (2.3)	
Low autonomy^a^			5.1 (2.6)				4.8 (2.8)	
High psychological demands^a^			5.3 (3.0)				5.2 (2.9)	
**Employment conditions**								
*Type of contract*				0.000				0.000
Permanent	10237	81.4	3.6		11169	82.2	3.3	
Non-permanent	1807	14.4	3.5		1846	13.6	3.1	
No contract	529	4.2	4.1		576	4.2	3.7	
*Income*				0.000				0.000
Sufficient household income	11073	84.6	3.4		11975	86.0	3.2	
Contributory earner with insufficient household income	760	5.8	4.3		327	2.3	3.9	
Main earner with insufficient household income	1258	9.6	4.6		1626	11.7	4.1	
*Irregular and/or unsocial working hours* ^*a*^			2.6 (2.2)				3.8 (2.5)	
*Employment status*								
Full-time	8822	67.4	3.6	0.001	12859	92.3	3.3	0.118
Part-time	3474	26.5	3.7		552	4.0	3.2	
Involuntary Part-time	795	6.1	3.8		517	3.7	3.4	
*Training*				0.154				0.001
Yes	6158	47.1	3.6		6143	44.2	3.3	
No	6911	52.9	3.6		7743	55.8	3.4	
**Employment relations**								
*Support from co-workers*				0.000				0.000
Yes	11939	92.6	3.6		13167	95.3	3.3	
No	952	7.4	4.2		648	4.7	3.9	
*Member of trade union*				0.424				0.357
Yes	2984	22.9	3.6		3277	23.6	3.3	
No	10073	77.1	3.6		10617	76.4	3.3	
**Poor mental well-being** ^**a**^			3.6 (2.0)				3.3 (1.9)	

^a^SE between parentheses.

### Mediation hypothesis

Table 2 shows social class, psychosocial work environment, employment conditions and relations to be related to poor mental well-being for the female sample. The reference model indicates that there are significant differences in mean poor mental well-being scores between the different age groups. Employed women between 30 and 49 years have a mean poor mental well-being score that is 0.246 points (SE = 0.044) higher compared to employees between 15 and 29 years of age. Employed women between 50 and 65 years have a mean poor mental well-being score that is 0.230 points (SE = 0.050) higher compared to the youngest age group.

**Table 2:** **Multilevel models for poor mental well-being in 14,164** employed ***women*** **aged 15–65 nested within 38 year-cohorts in 19 European countries (ESS 2004/5 and 2010)**

	Reference model b (SE)	Model 1 b (SE)	Model 2 b (SE)	Model 3 b (SE)	Model 4 b (SE)
**Fixed effects**					
Constant	3.368 (0.084)***	3.529 (0.096)***	3.352 (0.098)***	3.048 (0.096)***	3.480 (0.096)***
**Individual characteristics**					
*Age*					
15-29 years (ref.)					
30-49 years	0.246 (0.044)***	0.247 (0.044)***	0.248 (0.044)***	0.258 (0.045)***	0.240 (0.044)***
50 years and older	0.230 (0.050)***	0.214 (0.050)***	0.231 (0.049)***	0.266 (0.051)***	0.203 (0.050)***
*Social Class*					
Unskilled workers (ref.)					
Semi-skilled workers		-0.144 (0.056)**	-0.046 (0.056)	-0.099 (0.056)	-0.121 (0.056)*
Expert workers		-0.181 (0.059)**	0.022 (0.061)	-0.031 (0.060)	-0.146 (0.059)*
Non-expert supervisors		-0.252 (0.072)***	-0.064 (0.073)	-0.178 (0.072)*	-0.218 (0.072)**
Expert supervisors		-0.182 (0.069)**	0.042 (0.073)	-0.059 (0.071)	-0.140 (0.069)*
Non-expert managers		-0.212 (0.118)	0.024 (0.119)	-0.113 (0.117)	-0.187 (0.118)
Expert managers		-0.307 (0.100)**	-0.022 (0.103)	-0.158 (0.101)	-0.269 (0.100)**
**Psychosocial work environment**					
Low skill discretion			0.095 (0.008)***		
Low autonomy			0.033 (0.007)***		
High psychological demands			0.069 (0.006)***		
**Employment conditions**					
*Contract type*					
Permanent (ref.)					
Non-permanent				0.025 (0.049)	
No contract				0.175 (0.082)*	
*Income*					
Sufficient household income (ref.)					
Contributory earner with insufficient household income				0.785 (0.070)***	
Main earner with insufficient household income				0.891 (0.050)***	
*Irregular and/or unsocial hours*				0.057 (0.008)***	
*Employment status*					
Full-time (ref.)					
Part-time				0.071 (0.044)	
Involuntary part-time				0.162 (0.071)*	
*Lack of training*				0.058 (0.035)	
**Employment relations**					
*Lack of support*					0.490 (0.071)***
**Random effects** ^**a**^					
Level 1 variance	3.664 (0.044)	3.660 (0.044)	3.586 (0.043)	3.544 (0.042)	3.648 (0.044)
Level 2 variance	0.035 (0.015)	0.034 (0.015)	0.032 (0.014)	0.029 (0.013)	0.032 (0.014)
Level 3 variance	0.088 (0.037)	0.088 (0.036)	0.090 (0.037)	0.058 (0.026)	0.087 (0.036)
- 2 loglikelihood	-29338.4	-29329.3	-29185.3	-29097.5	-29305.4

*p ≤ 0.05; **p ≤ 0.01; ***p ≤ 0.001.

^*a*^ *All random effects are significantly different from zero at 95% confidence.*

In Model 1, including social class, expert managers on average report the lowest poor mental well-being scores compared to unskilled workers, while supervisors hold an intermediary position. When the indicators for the psychosocial work environment are added (Model 2), all significant associations between social class and mental well-being disappear. Low skill discretion (b = 0.095; SE = 0.008), low autonomy (b = 0.033; SE = 0.007) and high psychological demands (b = 0.069; SE = 0.006) are positively associated with poor mental well-being. Adding the indicators for employment conditions (Model 3), also discards all significant associations of mental well-being and social class, except for non-expert supervisors. They still report a poorer mental well-being score than unskilled workers (b = -0.178; SE = 0.072). Employed women without a contract report a mean poor mental well-being score that is 0.175 points (SE = 0.082) higher compared to female employees holding a permanent contract. Employees who perceive their household income as insufficient report a higher mean poor mental well-being score compared to employees who perceive their household income as sufficient. Employed women working involuntary part-time report a mean poor mental well-being score that is 0.162 points (SE = 0.071) higher compared to full-time workers. Poor mental well-being furthermore increases, as the degree of irregular and/or unsocial working hours increases (b = 0.057; SE = 0.008). When the indicator for employment relations is added (Model 4), all previous significant associations between poor mental well-being and social class remain. Employed women reporting a lack of co-worker support have a poor mental well-being score that is 0.490 points (SE = 0.071) higher compared to those not reporting a lack of support.

Table 3 shows social class, psychosocial work environment, employment conditions and relations to be related to poor mental well-being for the male sample. The reference model indicates significant differences in the mean poor mental well-being scores between the different age groups. Male employees between 30 and 49 years have a mean poor mental well-being score that is 0.294 points (SE = 0.041) higher compared to employees between 15 and 29 years. Male employees between 50 and 65 years have a mean poor mental well-being that is 0.221 points (SE = 0.046) higher compared to the youngest age group.

**Table 3:** **Multilevel models for poor mental well-being in 14,583** employed ***men*** **aged 15–65 nested within 38 year-cohorts in 19 European countries (ESS 2004/5 and 2010)**

	Reference model b (SE)	Model 1 b (SE)	Model 2 b (SE)	Model 3 b (SE)	Model 4 b (SE)
**Fixed effects**					
Constant	3.094 (0.086)***	3.194 (0.096)***	3.108 (0.097)***	2.809 (0.096)***	3.165 (0.096)***
**Individual characteristics**					
*Age*					
15-29 years (ref.)					
30-49 years	0.294 (0.041)***	0.305 (0.041)***	0.301 (0.041)***	0.270 (0.042)***	0.298 (0.041)***
50 years and older	0.221 (0.046)***	0.228 (0.046)***	0.248 (0.046)***	0.234 (0.047)***	0.217 (0.046)***
*Social Class*					
Unskilled workers (ref.)					
Semi-skilled workers		-0.108 (0.054)*	-0.052 (0.053)	-0.038 (0.054)	-0.096 (0.054)
Expert workers		-0.020 (0.061)	0.127 (0.062)*	0.144 (0.062)*	-0.007 (0.061)
Non-expert supervisors		-0.185 (0.059)**	-0.045 (0.060)	-0.098 (0.059)	-0.172 (0.059)**
Expert supervisors		-0.147 (0.065)*	0.038 (0.068)	-0.003 (0.067)	-0.123 (0.065)
Non-expert managers		-0.247 (0.091)**	-0.060 (0.092)	-0.130 (0.091)	-0.224 (0.091)*
Expert managers		-0.160 (0.077)*	0.040 (0.080)	-0.021 (0.079)	-0.138 (0.077)
**Psychosocial work environment**					
Low skill discretion			0.090 (0.008)***		
Low autonomy			0.034 (0.007)***		
High psychological demands			0.087 (0.006)***		
**Employment conditions**					
*Contract type*					
Permanent (ref.)					
Non-permanent				-0.045 (0.049)	
No contract				0.037 (0.075)	
*Income*					
Sufficient household income (ref.)					
Contributory earner with insufficient household income				0.619 (0.104)***	
Main earner with insufficient household income				0.818 (0.046)***	
*Irregular and/or unsocial hours*				0.039 (0.007)***	
*Employment status*					
Full-time (ref.)					
Part-time				-0.012 (0.081)	
Involuntary part-time				0.159 (0.082)	
*Lack of training*				0.053 (0.034)	
**Employment relations**					
*Lack of support*					0.536 (0.077)***
**Random effects** ^**a**^					
Level 1 variance	3.379 (0.040)	3.374 (0.040)	3.289 (0.039)	3.293 (0.039)	3.363 (0.039)
Level 2 variance	0.023 (0.011)	0.023 (0.011)	0.025 (0.012)	0.020 (0.010)	0.022 (0.011)
Level 3 variance	0.105 (0.040)	0.106 (0.040)	0.105 (0.040)	0.075 (0.029)	0.105 (0.039)
- 2 loglikelihood	-29612.5	-29602.9	-29419.7	-29421.1	-29578.5

*p ≤ 0.05; **p ≤ 0.01; ***p ≤ 0.001.

^*a*^ *All random effects are significantly different from zero at 95% confidence.*

The social class indicators are added in Model 1, showing that compared to unskilled workers, non-expert managers report the lowest mean poor mental well-being scores and supervisors hold an intermediary position. When the indicators for the psychosocial work environment are added (Model 2), all significant associations between poor mental well-being and the social class indicators disappear, except for expert workers. Low skill discretion (b = 0.090; SE = 0.008), low autonomy (b = 0.034; SE = 0.007) and high psychological demands (b = 0087; SE = 0.006) are positively associated with poor mental well-being. Adding the indicators for employment conditions to Model 1 (Model 3) discards all significant associations of mental well-being and social class, except for expert workers. Employees who perceive their household income as insufficient report a higher mean poor mental well-being score compared to employees who perceive their household income as sufficient. Furthermore, poor mental well-being increases as the degree of irregular and/or unsocial working hours increases (b = 0.039; SE = 0.007). When the indicator for employment relations is added to Model 1 (Model 4), nearly all associations between poor mental well-being and social class become statistically insignificant. Only non-expert supervisors and managers still report a poor mental well-being score that is lower compared to unskilled workers (a poor mental well-being score that is 0.172 (SE = 0.059) and 0.138 (SE = 0.077) points lower respectively). Employed men reporting a lack of co-worker support have a poor mental well-being score that is 0.536 points (SE = 0.077) higher compared to those not reporting a lack of support.

### Moderation hypothesis

Table 4 shows Model 2 and 3 extended by the interactions between social class and the indicators for the psychosocial work environment and employment conditions respectively. As the interactions between social class and the indicators of employment relations were not significant, results are not shown. Only significant interactions are presented. Both for men and women, there is effect modification between social class and some of the indicators of the psychosocial work environment and employment conditions. For instance, male expert supervisors and managers report a poorer mental well-being when exposed to high psychological demands, compared to male unskilled workers. Female expert workers report a poorer mental well-being when exposed to low skill discretion and high psychological demands, compared to female unskilled workers. Male non-expert managers report a poorer mental well-being when exposed to an insufficient income while being contributory earner, compared to male unskilled workers.

**Table 4:** **Summary of significant interaction effects in the multilevel models for poor mental well-being in European employees aged 15–65 nested within 38 year-cohorts in 19 European countries (ESS 2004/5 and 2010)**

Interactions with psychosocial work environment	Extended model 2 for employed women b (SE)	Extended model 2 for employed men b (SE)	Interactions with employment conditions	Extended model 3 for employed women b (SE)	Extended model 3 for employed men b (SE)
Semi-skilled workers x Low skill discretion	0.055 (0.023)*		Semi-skilled workers x Contr. earner w. in. income	-0.473 (0.188)*	
Semi-skilled workers x High psy. demands	0.042 (0.019)*		Semi-skilled workers x Lack of training	-0.238 (0.118)*	
Expert workers x Low skill discretion	0.059 (0.034)*		Expert workers x Main earner w. in. income	-0.495 (0.171)**	
Expert workers x High psy. demands	0.062 (0.020)**		Expert workers x Lack of training	-0.312 (0.125)*	
Non-expert managers x Low autonomy	-0.115 (0.047)*		Non-expert supervisors x Main earner w. in. income	-0.478 (0.209)*	
Non-expert managers x High psy. demands	0.088 (0.041)*		Non-expert managers x Involuntary part-time	1.489 (0.600)*	1.141 (0.509)*
Expert managers x Low autonomy	-0.133 (0.048)**		Non-expert managers x Lack of training	-0.696 (0.239)**	
Expert supervisors x High psy. demands		0.053 (0.24)*	Non-expert managers x Contr. earner w. in. income		0.571 (0.845)**
Expert managers x High psy. demands		0.080 (0.028)**			

*p ≤ 0.05; **p ≤ 0.01.

*Abbreviations: psy.: psychological; Contr.: Contributory; w. in. income: with insufficient household income.*

## Discussion

In this study, Wright’s relational social class scheme was used to map out structural social inequalities in employee mental well-being. In addition, indicators for the psychosocial work environment and employment quality accounted for the intermediary determinants of inequalities in mental well-being. We found social class inequalities in the mental well-being of the European working population: for both men and women non-supervisory non-managerial unskilled workers reported the worst mental well-being. Compared to unskilled workers, managers reported the best mental well-being, while supervisors held an intermediary position. As expected and in accordance with previous studies [18, 19], managers showed better mental well-being than the other class positions. Furthermore, our results showed that supervisors reported a better mental well-being than unskilled workers, implying that the location of supervisors within class relations does not result in the worst mental well-being in European employees.

In contrast to our expectations, we did not always find a better mental well-being in experts than non-experts. Among men, supervisors and managers with an expert skill level reported a worse mental well-being than supervisors and managers with lower skill levels. Among women, expert-level supervisors also reported a worse mental well-being than lower-skilled supervisors. Our findings are consistent with previous research [26] and research on status incongruence [39]. Expert supervisors and managers can be considered high-status congruents, that is workers who simultaneously hold a less exploited, more empowered class location on the organisational control dimension and on the skill dimension. According to Lundberg et al. [39] high-status congruent individuals show an elevated risk for experiencing shaming, that is the sensation of not being regarded and respected in the way one thinks one deserves to be. Shaming experiences are a primary producer of poor mental well-being [39]. Expert managers and supervisors are less exploited. Yet, they also occupy a “contradictory class location” [25]. They can still experience events of domination, for example being disciplined for a poor performance by their own superiors (their owners or their boards). These disciplinary measures are more likely to offend them and evoke feelings of shame, because of inadequate confirmation of their social location by others [39]. In other words, although expert supervisors and managers are less exploited compared to other employees, the external sanctions they do experience may have a larger impact on their well-being. The finding that occupying an expert location is not always protective of mental well-being is in line with Wright’s indicators of skills/credentials as a measure of social class, rather than with social stratification theory.

Our study also sheds some light on the mechanisms that mediate/moderate the relationship between social class and poor mental well-being among men and women. As regards the moderation hypothesis, both for employed men and women, there is effect modification between social class and some of the indicators of the psychosocial work environment and employment conditions, implying differential vulnerability. As regards the mediation hypothesis, we found, in accordance with previous research on self-reported health [18], that explaining the associations between social class and mental well-being involved different mediating factors for men and women. Our results showed that an unfavourable psychosocial work environment and low-quality employment conditions mediate the relation between social class and mental well-being for both men and women. However, low-quality employment relations only mediate the relation between social class and mental well-being for men. An explanation for this could be found in differences in social roles and in the different meaning of employment relations for men and women [40]. Although both men and women place high value on the employee and homemaker roles [41], typically, employment relations are of higher importance to men, than to women. Historically and culturally throughout the male breadwinner model, men attach more meaning to the labour role which is key to their identity, while the labour role of women is often secondary to their caring/parenting role. Yet, male non-expert supervisors and managers still experience better mental well-being than male unskilled workers, even after accounting for employment relations. An explanation for this might be that non-expert supervisors and managers reached their position by upward career promotion, rather than as a consequence of a high education. Upward mobility changes the social location of people, but not necessarily their values and expectations. On the contrary, values and expectations will often differ from those of the new socio-cultural context [39]. As a consequence, the expectations of non-expert supervisors and managers about the employment relations could still be similar to the expectations of the lower social classes, and thus be minified. Therefore, the employment relations may have no mediating effect on the relation between mental well-being and social class for these classes.

Interestingly, male expert workers reported the worst mental well-being after controlling for the adverse psychosocial work environment and employment conditions. This finding is consistent with the finding that negatively incongruent individuals report a higher frequency of poor mental well-being, compared to other groups [39]. Expert workers can be considered negatively incongruent, as they hold a more exploited, less empowered class location on the organisational control dimension and a less exploited, more empowered class location on the skill dimension.

Due to the use of secondary data, our study bears some limitations. First, our data are derived from a cross-sectional sample, so we cannot formally establish the causal direction of the relationships under study. Second, the ISCED-classification used to operationalize skills and expertise is only a proxy for the underlying theoretical concept, as skills and expertise may be recognised without being formally certified by an educational degree [25]. Including skills and expertise other than acquired through schooling might strengthen the observed theory. Third, the dataset used in this study is a pooled dataset containing data from 2004/2005 and 2010. However, as of 2008, an economic and social crisis has taken place in European countries. This economic crisis has shown to impact mental health and even suicide mortality [42, 43]. However, our models show a small between-year-within-country variance, indicating that the average mental well-being of employees in a certain country was not that different in 2004/5 compared to 2010.

The present study also has several strengths. First, a clear strength of this study is the use of proxies of relational indicators of social class. Our results underscore the importance of relational indicators of social class for assessing social inequalities in mental health of European employees. They provide a complementary approach to stratification indicators in social epidemiology and public health research. Second, although several studies have underscored that explaining social inequalities in health involves mediation and moderation mechanisms [22, 26, 27], few studies have investigated these mechanisms simultaneously.

## Conclusions

Social class is related to mental well-being in European employees. From a policy perspective, this study implies that better employee mental well-being and less social inequalities could be achieved by initiatives addressing the unequal social relations generated by structural positions in the labour process, such as regular meetings between representatives of employers and different groups of employees. Vertical bureaucracy, typically associated with Fordist systems, should be transformed into more horizontal organisational structures [44]. Moreover, our results underline the importance of the psychosocial work environment, of the employment conditions and relations for understanding social class inequalities in mental well-being and for improving the mental well-being of European employees.
